# Association between statin use and longitudinal changes in skeletal muscle mass: A population-based cohort study

**DOI:** 10.1371/journal.pone.0355202

**Published:** 2026-09-03

**Authors:** Jiye Lee, Suho Jun, Jun-Hyuk Lee, Jee Hye Han, Hyun Jung Kim

**Affiliations:** 1 Department of Rehabilitation Medicine, Nowon Eulji Medical Center, Eulji University School of Medicine, Seoul, Republic of Korea; 2 Department of Family Medicine, Nowon Eulji Medical Center, Eulji University School of Medicine, Seoul, Republic of Korea; University Hospital of Padova, ITALY

## Abstract

Statins are widely prescribed for cardiovascular risk reduction, yet concerns remain regarding their effects on skeletal muscle. Evidence on the longitudinal relationship between statin use and muscle mass remains inconsistent, particularly in population-based settings. Therefore, this study investigated the longitudinal association between statin use and changes in total and region-specific skeletal muscle mass in a population-based adult cohort. This single-center retrospective cohort study analyzed adults with repeated health examinations from 2021 to 2023. Skeletal muscle mass was assessed using bioelectrical impedance analysis, including upper limb lean mass (ULM), lower limb lean mass (LLM), appendicular lean mass (ALM), and skeletal muscle index (SMI). Statin use was determined from medical records and self-reported medication histories. Linear mixed-effects models with random intercepts helped evaluate longitudinal changes in muscle mass according to statin use. Models were sequentially adjusted for age and sex, and further for body mass index and diabetes mellitus. Among 2,300 participants, 334 (14.5%) were continuous statin users, who were older and had a higher prevalence of metabolic comorbidities than non-users. In unadjusted analyses, statin users exhibited significantly greater declines in LLM and ALM than non-users. These differences remained significant after adjustment for age and sex. However, after further adjustment for body mass index and diabetes mellitus, associations between statin use and changes in all muscle mass indices were no longer significant. No significant differences were observed for ULM or SMI in any model. In this longitudinal cohort, statin use was not independently associated with accelerated skeletal muscle mass decline after adjustment for key metabolic confounders. These findings suggest that underlying metabolic and clinical characteristics, rather than statin exposure, may play a more important role in longitudinal muscle mass changes.

## Introduction

Sarcopenia is a multifactorial condition characterized by a gradual, widespread skeletal muscle deterioration, leading to reduced muscle mass, functional capacity, and overall physical performance. Although commonly associated with aging, sarcopenia is influenced by comorbidities and lifestyle-related factors and is associated with adverse outcomes such as falls, disability, hospitalization, and mortality [1,2]. International consensus definitions, including the revised European Working Group on Sarcopenia in Older People and Asian Working Group for Sarcopenia, identify low muscle mass as a key diagnostic criterion and describe sarcopenia as cumulative muscle deterioration throughout adulthood [1,2]. In South Korea, rapid population aging has made sarcopenia a major public health concern, with a pooled prevalence of approximately 13% among adults aged ≥65 years, comparable to global estimates of 10–16% among older populations [3–5].

Statins are widely prescribed for primary and secondary cardiovascular disease prevention, and effectively reduce atherosclerotic cardiovascular events [6,7]. Despite their overall favorable cardiovascular risk–benefit profile, statins have been associated with muscle-related symptoms, ranging from mild myalgia to rare severe myopathy and rhabdomyolysis [8,9]. Beyond clinically recognized muscle symptoms, experimental studies suggest that statins may influence skeletal muscle via mitochondrial dysfunction, electron transport chain inhibition, and altered myogenic differentiation and regeneration, raising theoretical concerns about potential long-term effects on muscle health [10–12].

Epidemiologic evidence on the association between statin use and sarcopenia or muscle function remains inconsistent. Some observational studies report adverse muscle-related or functional outcomes among statin users, particularly among vulnerable populations such as older adults or patients with stroke [13–15]. However, a recent large-scale longitudinal cohort study using contemporary diagnostic criteria found no significant association between long-term statin use and incident sarcopenia in community-dwelling adults [16].

Collectively, evidence linking statin use and sarcopenia remains inconclusive owing to substantial heterogeneity in study designs, populations, outcome definitions, and analytical approaches. In particular, many studies rely on cross-sectional data or specific clinical subgroups, limiting causal inference and generalizability. Moreover, few investigations have examined longitudinal region-specific muscle mass changes while adequately accounting for key metabolic confounders.

In this context, this study examined longitudinal associations between statin use and changes in total and region-specific muscle mass in a large adult cohort, accounting for key demographic and metabolic factors. By addressing key methodological limitations of prior studies, this study provides novel longitudinal evidence that helps clarify inconsistent findings regarding the relationship between statin use and skeletal muscle mass.

## Methods

### Study design and participants

This retrospective longitudinal cohort study analyzed data from adults who voluntarily underwent repeated health check-ups at Nowon Eulji Medical Center between 01 January 2021 and 31 December 2023. Participants aged ≥18 years old who completed at least two health check-ups including bioelectrical impedance analysis (BIA) were eligible for inclusion. Health check-ups were conducted at variable intervals, depending on individual participation patterns.

**Fig 1:**
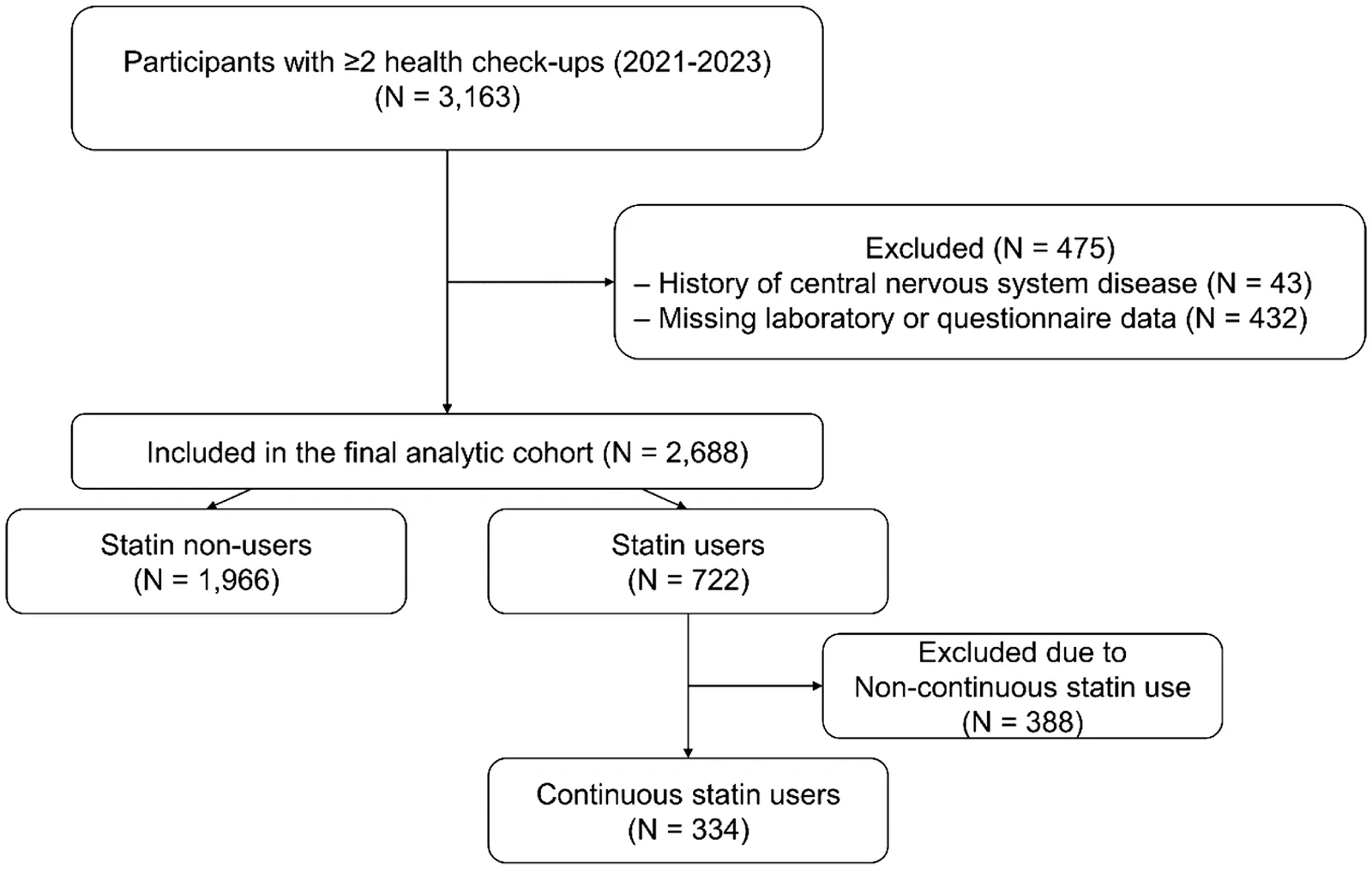
Flowchart of participant selection and classification based on statin use.

Participants were excluded if they had a history of central nervous system diseases, including stroke, brain tumor, spinal cord disease, or other neurological disorders affecting muscle mass, or if they had missing laboratory or questionnaire data. After applying eligibility criteria, the final analytic cohort included participants with complete longitudinal muscle mass measurements. The participant selection process is illustrated in Fig 1.

Baseline demographic, clinical, and laboratory, comorbidity, and health-related questionnaire data were collected at the first health examination.

The study protocol was approved by the Institutional Review Board of Nowon Eulji Medical Center (IRB No. 2024-01-008), with the requirement for informed consent waived owing to the retrospective nature of the study. The data were first accessed for research purposes on 24 January 2024. The authors had access to identifiable information during data extraction; however, all data were fully de-identified prior to statistical analysis.

### Exposure: Statin use

Statin use was defined based on medication records obtained from health check-up questionnaires and electronic medical records. Participants were classified into statin users and non-users.

Continuous statin users were defined as individuals who consistently reported statin use at all health check-ups or had corresponding prescription records during follow-up to minimize exposure misclassification and ensure sustained statin exposure over time. Statin use was treated as a fixed exposure variable, and only participants with consistent statin use throughout follow-up were included in the statin user group.

Statin exposure was defined as continued statin use at each recorded health examination throughout the observation period. Although cumulative duration was not modeled as a continuous variable, this definition ensured sustained exposure during follow-up.

Statins included commonly prescribed agents such as atorvastatin, rosuvastatin, pravastatin, pitavastatin, and simvastatin.

### Outcome measures

Muscle mass was assessed using bioelectrical impedance analysis (InBody system; InBody Co., Ltd., Seoul, Republic of Korea) during each health check-up. The following outcomes were evaluated:

- Upper limb lean mass (ULM)
- Lower limb lean mass (LLM)
- Appendicular lean mass (ALM)
- Skeletal muscle index (SMI), calculated as appendicular lean mass divided by height squared (kg/m²)

All measurements were obtained once per health check-up with participants standing under standardized conditions, including light clothing and removal of metal accessories, according to the manufacturer’s protocol.

### Covariates

Covariates were selected a priori based on their established associations with both statin use and skeletal muscle mass, as well as their potential to confound the relationship between statin exposure and longitudinal muscle changes. The following covariates were included in the primary adjusted analyses:

- Age (years)
- Sex
- Body mass index (BMI, kg/m²)
- Diabetes mellitus (yes/no)

Additional variables, including serum albumin level, healthy dietary score, and hypertension, were selected based on their known clinical relevance to skeletal muscle mass and metabolic health. These variables were included in sensitivity analyses to further evaluate the robustness of the primary findings. These variables were not included in the primary model to avoid potential overadjustment or multicollinearity with key metabolic factors such as BMI and diabetes mellitus.

### Statistical analyses

Baseline demographic and clinical characteristics were summarized and compared between statin users and non-users using independent t-tests or Mann–Whitney U tests for continuous variables and chi-square tests for categorical variables.

Longitudinal muscle mass changes were analyzed using linear mixed-effects models appropriate for repeated measurements with a minimum of two assessments per participant. Models included a random intercept specified for each individual and an autoregressive covariance structure of order 1 (AR(1)) to account for correlations among repeated observations.

The fixed-effects structure included statin use, time, and their interaction term, with the statin use-time interaction serving as the primary parameter of interest to assess differential muscle mass trajectories according to statin exposure. Models were constructed sequentially, beginning with an unadjusted model, followed by adjustment for age and sex, and finally a fully adjusted model incorporating age, sex, body mass index, and diabetes mellitus. To address potential residual confounding, additional sensitivity analyses were performed with further adjustment for albumin, healthy dietary score, and hypertension.

Subgroup analyses were conducted among participants with follow-up duration ≥1 year and those aged ≥60 years. These analyses were performed to assess the robustness of the findings and account for variability in follow-up duration.

Participants with non-continuous statin use were excluded from the primary analysis to ensure consistent exposure classification. To evaluate potential selection bias, including potential depletion of susceptibles, baseline characteristics of non-continuous statin users were compared with those of continuous users and non-users.

Because ULM, LLM, ALM, and SMI represent biologically related and partially overlapping measures of skeletal muscle mass rather than independent endpoints, formal correction for multiple comparisons was not applied. Results were interpreted with caution, considering consistency across outcomes and models.

Estimated marginal means were derived to visualize longitudinal muscle mass patterns according to statin use. Statistical significance was defined as a two-sided p-value <0.05. Analyses were conducted using IBM SPSS Statistics (version 31; IBM Corp., Armonk, NY, USA).

## Results

### Baseline characteristics

A total of 2,300 participants were included in the final longitudinal analysis, consisting of 1,966 (85.5%) statin non-users and 334 (14.5%) continuous statin users. Participants with non-continuous statin use were excluded to ensure clear exposure classification. Baseline characteristics of the final analytic cohort are presented in Table 1. The participant selection process, including exclusions owing to non-continuous statin use, is illustrated in Fig 1. Baseline characteristics of non-continuous statin users and pairwise comparisons are provided in S1 and S2 Tables.

**Table 1:** Baseline characteristics of the study population according to statin use.

Variable	Non-users (n = 1,966)	Statin users (n = 334)	p-value
**Demographics**			
Male sex, n (%)	1104 (56.2)	213 (63.8)	**0.009‡**
Female sex, n (%)	862 (43.8)	121 (36.2)	**0.009‡**
Age, years	45.4 ± 9.9	54.7 ± 8.6	**<0.001†**
Height, cm	168.5 ± 8.3	167.2 ± 8.6	**0.013†**
Weight, kg	67.6 ± 13.2	70.0 ± 13.2	**0.002†**
Body mass index, kg/m²	23.7 ± 3.4	24.9 ± 3.3	**<0.001†**
Follow-up duration, years	1.71 (1.02-2.00)	1.85 (1.09-2.02)	**0.013§**
Number of visits	2 (2-3)	2 (2-3)	0.210§
**Body composition**			
Body fat mass, kg	18.7 ± 6.2	20.6 ± 6.2	**<0.001†**
Upper limb lean mass, kg	5.12 ± 1.53	5.31 ± 1.46	**0.025†**
Lower limb lean mass, kg	15.56 ± 3.38	15.56 ± 3.49	0.989†
Appendicular lean mass, kg	20.68 ± 4.85	20.87 ± 4.90	0.506†
Skeletal muscle index, kg/m²	7.18 ± 1.13	7.36 ± 1.12	**0.010†**
**Laboratory parameters**			
Albumin, g/dL	4.50 ± 0.25	4.61 ± 0.27	**<0.001†**
Total cholesterol, mg/dL	200.0 ± 33.3	170.8 ± 41.7	**<0.001†**
Triglycerides, mg/dL	102 (72–149)	115 (85–162)	**<0.001§**
HDL cholesterol, mg/dL	55.9 ± 15.5	51.9 ± 12.5	**<0.001†**
LDL cholesterol, mg/dL	127.2 ± 32.3	101.0 ± 38.8	**<0.001†**
HbA1c, %	5.54 ± 0.57	6.14 ± 0.87	**<0.001†**
White blood cell count, × 10³/µL	5.24 ± 1.47	5.69 ± 2.04	**<0.001†**
Hemoglobin, g/dL	14.52 ± 1.47	14.60 ± 1.38	0.306†
CRP, mg/dL	0.062 (0.033–0.127)	0.055 (0.029–0.110)	**0.040§**
**Questionnaire variables**			
PHQ-9 score	2 (0–5)	1.5 (0–4)	**0.010§**
GAD-7 score	1 (0–4)	0 (0–3)	**0.005§**
Healthy dietary score	−7 (−10 to −4)	−5 (−8 to −2)	**<0.001§**
Current smoker, n (%)	426 (21.7)	77 (23.1)	0.571‡
Alcohol consumption, n (%)	1612 (82.0)	261 (78.1)	0.094‡
Physical activity, n (%)			0.912‡
Level 1	1608 (81.8)	275 (82.3)	
Level 2	310 (15.8)	53 (15.9)	
Level 3	41 (2.1)	5 (1.5)	
Level 4	7 (0.4)	1 (0.3)	
**Comorbidities**			
Heart disease, n (%)	22 (1.1)	13 (3.9)	**<0.001‡**
Hypertension, n (%)	189 (9.6)	157 (47.0)	**<0.001‡**
Diabetes mellitus, n (%)	47 (2.4)	97 (29.0)	**<0.001‡**
Dyslipidemia, n (%)	5 (0.3)	182 (54.5)	**<0.001‡**

Physical activity was categorized into four ordinal levels, with Level 1 indicating the lowest and Level 4 the highest activity level.

Values are presented as the mean ± standard deviation or median (interquartile range), as appropriate.

† Continuous variables were compared using independent or Welch’s t-tests when the assumption of equal variances was violated.

§ Skewed continuous variables were compared using the Mann–Whitney U test.

‡ Categorical variables were compared using the chi-square or Fisher’s exact tests, as appropriate.

Compared with statin non-users, statin users were significantly older (p < 0.001) and had higher body weight (p = 0.002), body fat mass (p < 0.001), and body mass index (BMI; p < 0.001). Statin users also showed a higher prevalence of metabolic comorbidities, including diabetes mellitus (p < 0.001) and dyslipidemia (p < 0.001), and differed in sex distribution (p = 0.009). Follow-up duration was slightly longer in statin users (p = 0.013), whereas the number of visits was comparable between the groups (p = 0.210). Other baseline characteristics, including laboratory parameters, cardiovascular comorbidities, questionnaire-based assessments, and baseline measures of ULM, LLM, ALM, and SMI, are presented in Table 1.

In comparisons including non-continuous statin users, non-continuous users were generally younger and had lower albumin levels but less favorable lipid profiles, characterized by higher total cholesterol, triglyceride, and LDL cholesterol levels, compared with those of continuous statin users (all p < 0.05). They also had lower healthy dietary scores and a lower prevalence of hypertension, diabetes mellitus, and dyslipidemia (all p < 0.05; S1 and S2 Tables).

### Longitudinal changes in muscle mass: unadjusted analysis

In unadjusted linear mixed-effects models, significant statin-by-time interaction terms were observed for LLM (−0.081 vs. −0.045 kg/year; Δβ = +0.036 kg/year, interaction p = 0.038) and ALM (−0.084 vs. −0.034 kg/year; Δβ = +0.050 kg/year, interaction p = 0.042) (Table 2), indicating that longitudinal changes differed according to statin use. Specifically, both LLM and ALM declined significantly over time among statin users, with significantly steeper declines than in non-statin users.

**Table 2:** Time-related changes in skeletal muscle outcomes and between-group differences based on statin use (linear mixed models).

Outcome	Statin users slope (95% CI)	p for time	Δ slope (Non-statin − Statin) (95% CI)	p for interaction	Non-statin users slope
**Upper limb lean mass**	−0.003 (−0.021 to 0.015)	0.753	+0.014 (−0.006 to 0.034)	0.168	+0.011
**Lower limb lean mass**	−0.081 (−0.112 to −0.050)	<0.001	+0.036 (0.002 to 0.070)	**0.038**	−0.045
**Appendicular lean mass**	−0.084 (−0.128 to −0.039)	<0.001	+0.050 (0.002 to 0.099)	**0.042**	−0.034
**Skeletal muscle index (SMI)**	−0.013 (−0.027 to 0.001)	0.078	+0.012 (−0.003 to 0.028)	0.121	−0.001

Values are presented as β coefficients with 95% confidence intervals derived from linear mixed-effects models with random intercepts for participants.

β coefficients represent estimated annual rate of change (slope) in each outcome.

Statin users served as the reference group.

The time coefficient represents the rate of change over time in statin users.

Δ slope represents the between-group difference in the rate of change (non-statin minus statin).

Non-statin users slope is presented as a point estimate derived from the linear combination of fixed-effect coefficients.

A positive Δ slope indicates a less steep decline (or relative preservation) among non-statin users compared with statin users.

Statistically significant p-values (p < 0.05) are shown in bold.

In contrast, no significant statin-by-time interactions were observed for ULM or SMI, indicating similar longitudinal trajectories between statin users and non-users for these outcomes.

### Adjusted longitudinal analysis

After adjustment for age and sex, the statin-by-time interaction term was not significant for ULM or SMI (Table 3). In contrast, significant statin-by-time interactions were observed for LLM (−0.021 vs. + 0.015 kg/year; Δβ = +0.036 kg/year, interaction p = 0.040) and ALM (−0.009 vs. + 0.040 kg/year; Δβ = +0.049 kg/year, interaction p = 0.046), indicating differential longitudinal changes between statin users and non-users after adjustment for age and sex.

**Table 3:** Time-related changes in skeletal muscle outcomes and between-group differences based on statin use (linear mixed models adjusted for age and sex).

Outcome	Statin users slope (95% CI)	p for time	Δ slope (Non-statin − Statin) (95% CI)	p for interaction	Non-statin users slope
**Upper limb lean mass**	+0.013 (−0.006 to 0.031)	0.174	+0.013 (−0.007 to 0.033)	0.197	+0.026
**Lower limb lean mass**	−0.021 (−0.054 to 0.011)	0.194	+0.036 (0.002 to 0.070)	**0.040**	+0.015
**Appendicular lean mass**	−0.009 (−0.055 to 0.037)	0.695	+0.049 (0.001 to 0.098)	**0.046**	+0.040
**Skeletal muscle index (SMI)**	−0.004 (−0.019 to 0.010)	0.559	+0.012 (−0.004 to 0.027)	0.133	+0.008

Values are presented as β coefficients with 95% confidence intervals derived from linear mixed models with random intercepts for participants.

Models were adjusted for age and sex.

β coefficients represent estimated annual rate of change (slope) in each outcome.

Statin users served as the reference group.

The time coefficient represents the rate of change over time in statin users.

Δ slope represents the between-group difference in the rate of change (non-statin minus statin).

Non-statin users slope is presented as a point estimate derived from the linear combination of fixed-effect coefficients.

A positive Δ slope indicates a less steep decline (or relative preservation) among non-statin users compared with statin users.

Statistically significant p-values (p < 0.05) are shown in bold.

In the fully adjusted model including age, sex, BMI, and diabetes mellitus, the statin-by-time interaction term was not statistically significant for any muscle mass outcome (ULM, LLM, ALM, or SMI; all p > 0.05) (Table 4), indicating no evidence of differential longitudinal muscle mass changes according to statin use after full adjustment.

**Table 4:** Time-related changes in skeletal muscle outcomes and between-group differences based on statin use (linear mixed models adjusted for age, sex, body mass index, and diabetes mellitus).

Outcome	Statin users slope (95% CI)	p for time	Δ slope (Non-statin − Statin) (95% CI)	p for interaction	Non-statin users slope
**Upper limb lean mass**	+0.024 (0.009 to 0.040)	0.002	+0.001 (−0.016 to 0.017)	0.938	+0.025
**Lower limb lean mass**	−0.006 (−0.035 to 0.024)	0.695	+0.019 (−0.012 to 0.050)	0.235	+0.013
**Appendicular lean mass**	+0.018 (−0.021 to 0.057)	0.376	+0.020 (−0.021 to 0.062)	0.341	+0.038
**Skeletal muscle index (SMI)**	+0.006 (−0.006 to 0.018)	0.346	+0.001 (−0.012 to 0.014)	0.855	+0.007

Values are presented as β coefficients with 95% confidence intervals derived from linear mixed models with random intercepts for participants.

Models were adjusted for age, sex, body mass index, and diabetes mellitus.

β coefficients represent estimated annual rate of change (slope) in each outcome.

Statin users served as the reference group.

The time coefficient represents rate of change over time in statin users.

Δ slope represents the between-group difference in the rate of change (non-statin minus statin).

Non-statin users slope is presented as a point estimate derived from the linear combination of fixed-effect coefficients.

A positive Δ slope indicates a less steep decline (or relative preservation) among non-statin users compared with statin users.

Statistically significant p-values (p < 0.05) are shown in bold.

In additional sensitivity analyses further adjusting for albumin level, healthy dietary score, and hypertension, the statin-by-time interaction remained non-significant across all muscle mass outcomes (Table 5), supporting the robustness of the primary findings.

**Table 5:** Time-related changes in skeletal muscle outcomes and between-group differences based on statin use (linear mixed models adjusted for age, sex, body mass index, diabetes mellitus, albumin, healthy dietary score and hypertension).

Outcome	Statin users slope (95% CI)	p for time	Δ slope (Non-statin − Statin) (95% CI)	p for interaction	Non-statin users slope
**Upper limb lean mass**	+0.027 (−0.001 to 0.055)	0.062	−0.008 (−0.038 to 0.022)	0.604	+0.019
**Lower limb lean mass**	−0.019 (−0.058 to 0.020)	0.352	+0.025 (−0.017 to 0.067)	0.237	+0.006
**Appendicular lean mass**	+0.022 (−0.024 to 0.067)	0.346	+0.003 (−0.045 to 0.051)	0.910	+0.025
**Skeletal muscle index (SMI)**	−0.003 (−0.027 to 0.021)	0.819	−0.002 (−0.028 to 0.025)	0.901	−0.005

Values are presented as β coefficients with 95% confidence intervals derived from linear mixed models with random intercepts for participants.

Models were adjusted for age, sex, body mass index, diabetes mellitus, albumin, healthy dietary score and hypertension.

β coefficients represent estimated annual rate of change (slope) in each outcome.

Statin users served as the reference group.

The time coefficient represents rate of change over time in statin users.

Δ slope represents the between-group difference in the rate of change (non-statin minus statin).

Non-statin users slope is presented as a point estimate derived from the linear combination of fixed-effect coefficients.

A positive Δ slope indicates a less steep decline (or relative preservation) among non-statin users compared with statin users.

Statistically significant p-values (p < 0.05) are shown in bold.

### Estimated marginal means

**Fig 2:**
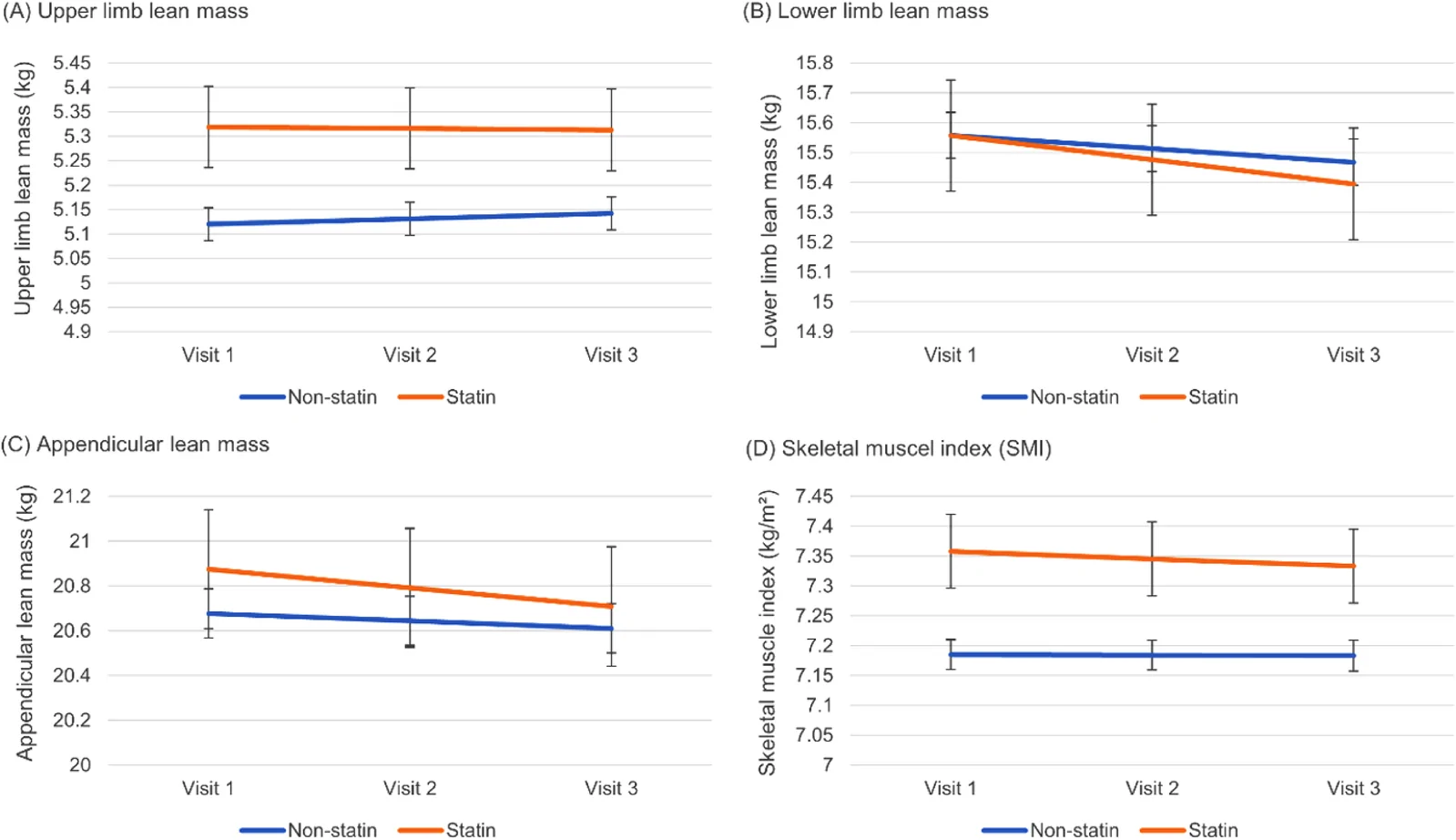
Estimated marginal mean trajectories of lean muscle mass based on statin use (unadjusted model). Panels show (A) upper limb lean mass, (B) lower limb lean mass, (C) appendicular lean mass, and (D) skeletal muscle index (SMI). Error bars indicate standard error.

**Fig 3:**
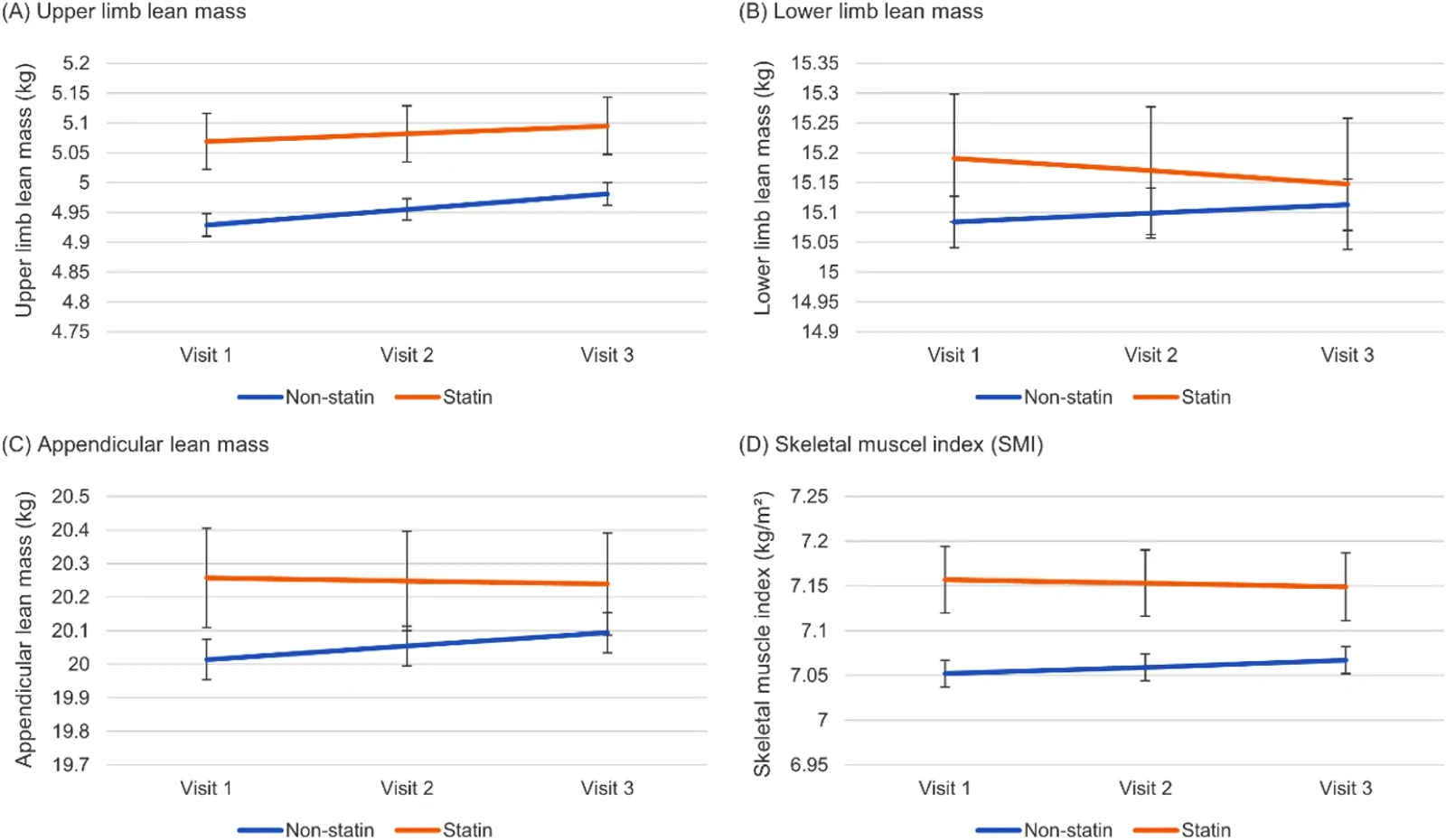
Estimated marginal mean trajectories of lean muscle mass based on statin use (adjusted for age and sex). Panels show (A) upper limb lean mass, (B) lower limb lean mass, (C) appendicular lean mass, and (D) skeletal muscle index (SMI). Estimated marginal means were derived from linear mixed models adjusted for age and sex. Error bars indicate standard error.

**Fig 4:**
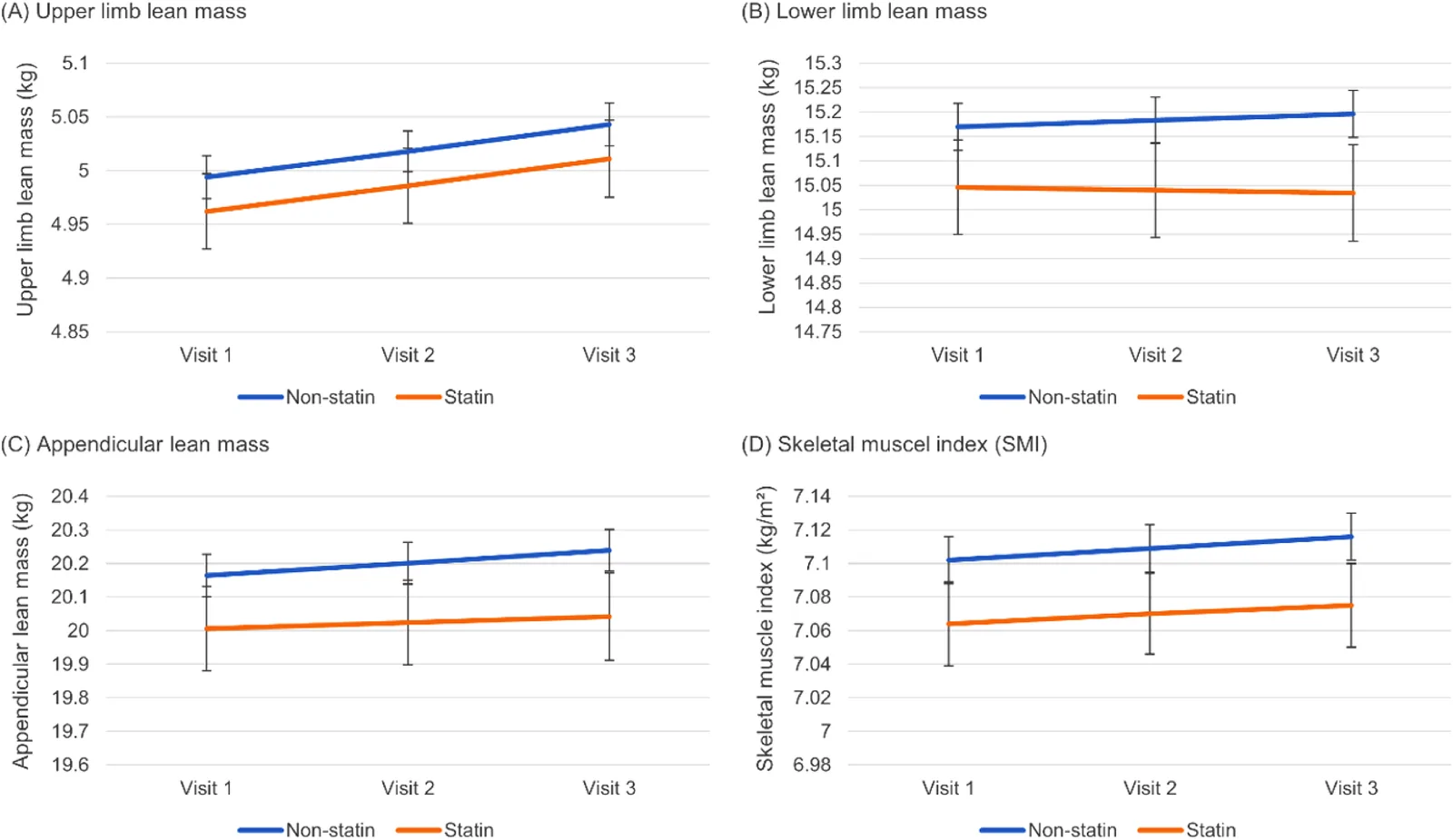
Estimated marginal mean trajectories of lean muscle mass based on statin use (adjusted for age, sex, body mass index, and diabetes mellitus). Panels show (A) upper limb lean mass, (B) lower limb lean mass, (C) appendicular lean mass, and (D) skeletal muscle index (SMI). Estimated marginal means were derived from linear mixed models adjusted for age, sex, body mass index, and diabetes mellitus. Error bars indicate standard error.

Estimated marginal means derived from the linear mixed-effects models are presented in Figs 2–4. In unadjusted models, estimated trajectories over time showed decreases in LLM and ALM in both groups, with differing rates of decline between statin users and non-users.

After adjustment for age and sex, differences in estimated LLM and ALM trajectories persisted, whereas no clear time-related patterns were observed for ULM or SMI. In the fully adjusted model including age, sex, BMI, and diabetes mellitus, estimated trajectories for statin users and non-users showed no apparent divergence over time across all muscle mass outcomes.

### Subgroup analyses

In subgroup analyses, no statistically significant statin-by-time interactions were observed in participants with follow-up duration ≥1 year (S3 Table) or in those aged ≥60 years (S4 Table) across all models.

## Discussion

### Principal findings

Uncertainty remains regarding the long-term effects of statin use on skeletal muscle mass, particularly with respect to longitudinal and region-specific changes. In this longitudinal cohort study, statin users exhibited steeper declines in lower limb and appendicular lean mass in unadjusted analyses. These associations persisted after adjustment for age and sex; however, they were attenuated and no longer significant after further adjustment for BMI and diabetes mellitus, suggesting that the observed differences in muscle mass trajectories were more closely related to underlying metabolic and clinical characteristics than to statin use itself.

Additional sensitivity analyses incorporating albumin, healthy dietary score, and hypertension yielded consistent findings, further supporting the robustness of the primary results. Moreover, subgroup analyses restricted to participants with longer follow-up duration (≥1 year) and those aged ≥60 years demonstrated no significant differences in longitudinal muscle mass changes according to statin use.

Comparisons including non-continuous statin users showed distinct baseline characteristics; however, these individuals did not exhibit features suggestive of a more vulnerable subgroup, reducing the likelihood that exclusion of non-continuous users substantially biased the primary findings.

### Comparison with previous studies

Previous studies reporting adverse statin-related muscle outcomes have often focused on high-risk populations, such as older adults or patients with stroke, who have more prevalent baseline muscle vulnerability and disease-related functional impairments [13–15]. In such populations, associations between statin use and muscle decline may reflect underlying metabolic and functional vulnerability rather than direct pharmacological effects of statins.

Consistent with this interpretation, differences in lower limb and appendicular lean mass trajectories observed in unadjusted and age- and sex-adjusted models were substantially attenuated after additional adjustment for body mass index and diabetes mellitus. This pattern suggested that metabolic and clinical characteristics—rather than statin exposure—may account for much of the apparent longitudinal muscle decline. Diabetes mellitus is consistently linked to accelerated skeletal muscle loss and increased sarcopenia risk, suggesting that baseline and ongoing metabolic dysregulation can dominate longitudinal muscle trajectories independent of statin use [17–19]. Similarly, longitudinal evidence indicates that certain BMI/weight trajectories—particularly those reflecting obesity or adverse weight-change patterns—are associated with greater declines in lean mass and physical function, highlighting the central role of metabolic health in shaping age-related muscle composition changes [20].

Diabetes and excess adiposity are mechanistically linked to metabolic dysregulation, including insulin resistance and ectopic lipid accumulation, which may adversely affect skeletal muscle metabolism. These metabolic disturbances are thought to disrupt the balance between muscle protein synthesis and degradation, predisposing individuals to progressive muscle loss under chronic metabolic stress [21]. Additionally, insulin resistance-related mitochondrial dysfunction impairs skeletal muscle oxidative capacity and energy metabolism, further contributing to muscle deterioration [22].

In this context, adjustment for BMI and diabetes in this study likely accounted for a substantial proportion of the metabolic burden underlying muscle loss, attenuating the apparent association between statin use and longitudinal muscle mass changes. These findings were further supported by sensitivity analyses incorporating additional metabolic and nutritional covariates, as well as subgroup analyses restricted to participants with longer follow-up duration and older age, which consistently showed no significant association between statin use and longitudinal muscle changes. Consistent with this interpretation, a large community-based cohort study using robust analytic approaches found no increased risk of sarcopenia or functional decline associated with long-term statin use after adjustment for key confounders [16].

### Clinical implications

Clinically, these findings do not provide evidence that statin use is associated with progressive skeletal muscle loss in this population. Instead, the observed patterns suggest that differences in underlying metabolic and clinical characteristics may play a more important role in longitudinal muscle changes than statin exposure itself.

These results highlight the importance of considering overall metabolic health when evaluating muscle outcomes in individuals receiving statin therapy. In particular, factors such as obesity and diabetes may substantially influence longitudinal muscle trajectories and should be addressed as part of comprehensive risk assessment.

While these findings are consistent with the absence of a significant association between statin use and muscle mass decline in this cohort, they should be interpreted cautiously. Further studies with longer follow-up and more detailed exposure characterization are warranted to confirm these observations. In this context, clinical decisions regarding statin therapy should continue to be individualized based on overall cardiovascular risk and patient characteristics.

### Strengths and limitations

The strengths of this study included its longitudinal design with repeated measurements and use of linear mixed-effects models to robustly assess within-individual muscle mass changes over time. Analysis of regional muscle compartments, including upper and lower limb lean mass, enabled a more detailed characterization of muscle aging patterns than reliance on composite indices such as skeletal muscle index. In addition, this study was conducted in a large, community-based population undergoing routine health check-ups, rather than in selected high-risk groups. This design enhances the generalizability of our findings and allows for the statin-related muscle change evaluation across a broad metabolic spectrum. Notably, associations between statin use and muscle loss were largely attenuated after adjustment for key metabolic factors, suggesting that underlying metabolic vulnerability rather than statin exposure may primarily drive longitudinal muscle decline. Together, these strengths provide clinically relevant insights into the relationship between statin use and muscle health and support a more nuanced interpretation of muscle-related outcomes in statin-treated patients.

Several limitations of this study should be acknowledged. First, this single-center, retrospective design limits causal inference, and the relatively short and variable follow-up duration across participants may not fully capture long-term age-related muscle loss trajectories. Although linear mixed-effects models allow for the inclusion of irregularly spaced repeated measurements, participants with shorter follow-up may have contributed limited information on longitudinal muscle changes. Second, although the overall sample size was relatively large, the proportion of statin users was smaller than that of non-users. Based on post-hoc power analyses, the minimum detectable effect size corresponded to approximately 0.022 kg/year for upper limb lean mass, 0.045 kg/year for lower limb lean mass, 0.059 kg/year for appendicular lean mass, and 0.017 kg/m²/year for skeletal muscle index. These estimates suggest that the study was adequately powered to detect small-to-moderate effect sizes; however, smaller differences in longitudinal muscle mass trajectories may have remained undetected. Therefore, the absence of statistically significant associations in fully adjusted models should be interpreted with caution. Third, residual confounding cannot be entirely excluded. Although key variables including age, sex, body mass index, and diabetes mellitus were included in the primary models, and additional sensitivity analyses were further adjusted for albumin level, healthy dietary score, and hypertension, other potential confounders may not have been fully captured. In particular, physical activity was assessed using a self-reported categorical measure based on subjective intensity (light, moderate, high, and very high intensity), with limited variability across participants, which may have reduced the ability to adequately control for its influence on muscle mass. Therefore, residual confounding related to physical activity may remain. Fourth, participants with non-continuous statin use were excluded to ensure consistent exposure classification. Although supplementary analyses did not indicate substantial differences, the possibility of selection bias, including depletion of susceptibles, cannot be entirely excluded. Fifth, statin exposure was derived from medical records and self-reported medication histories, and detailed information on statin type, dose, duration, and intensity was unavailable. Given that prior studies have demonstrated heterogeneity in muscle-related effects according to statin potency and formulation, the lack of detailed statin data (e.g., type and dose) may have contributed to exposure misclassification and attenuation of observed associations, potentially obscuring differential effects across statin subtypes [9]. Sixth, muscle mass was assessed using bioelectrical impedance analysis rather than dual-energy X-ray absorptiometry, which may be more susceptible to hydration status and device-specific variability [23]. Nevertheless, bioelectrical impedance analysis is widely used in large-scale epidemiologic studies and routine health screening settings and is considered an acceptable method for skeletal muscle mass assessment, including within the diagnostic framework proposed by the Asian Working Group for Sarcopenia [2,24]. However, potential measurement variability may have influenced the detection of subtle differences in longitudinal muscle mass changes. Finally, objective measures of muscle strength and physical performance were unavailable. Although current consensus definitions emphasize muscle strength and functional performance as core sarcopenia components [1,2], muscle mass remains a fundamental structural determinant of musculoskeletal and metabolic health. Muscle mass changes may accompany functional decline; however, muscle mass alone may not fully capture clinically meaningful functional outcomes [25]. Therefore, the absence of strength and performance measures limits the ability to fully interpret the clinical significance of the observed muscle mass changes. In addition, healthy user bias may have influenced the findings, as individuals undergoing routine health check-ups and maintaining consistent statin use may exhibit more favorable health behaviors and adherence patterns, compared to the general population, which could independently affect muscle mass trajectories. This may have attenuated the observed associations, potentially leading to an underestimation of any adverse effects of statin use on muscle mass. Therefore, these findings should be interpreted with caution, particularly when generalizing to higher-risk clinical populations.

## Conclusions

In this longitudinal cohort, statin use was not independently associated with accelerated skeletal muscle mass decline after adjustment for key metabolic confounders. Although greater muscle loss was observed among statin users in unadjusted analyses, these differences were attenuated after accounting for body mass index and diabetes mellitus, suggesting that underlying metabolic and clinical characteristics may contribute to the observed differences.

These findings suggest that statin use was not associated with significant differences in longitudinal muscle mass trajectories in this population. However, these results should be interpreted with caution, as the absence of a statistically significant association does not exclude the possibility of a true effect.

Further research is needed to confirm these findings and to better define the relationship between statin use and longitudinal muscle changes.

## Supporting information

S1 Table Baseline characteristics of the study population according to statin use (non-users, continuous users, and non-continuous users). (DOCX)

S2 Table Pairwise comparisons of baseline characteristics of the study population according to statin use (non-users, continuous users, and non-continuous users). (DOCX)

S3 Table Time-related changes in skeletal muscle outcomes and between-group differences based on statin use (linear mixed models, participants with follow-up ≥1 year; total n = 1789, statin users = 275, non-statin users = 1514). (DOCX)

S4 Table Time-related changes in skeletal muscle outcomes and between-group differences based on statin use (linear mixed models, participants aged ≥60 years; total n = 189, statin users = 77, non-statin users = 112). (DOCX)
