# Supplementary material for: Association between statin use and longitudinal changes in skeletal muscle mass: A population-based cohort study
Source: PLoS One. 2026 Sep 3;21(9):e0355202. doi: 10.1371/journal.pone.0355202 (PMC13541165; doi:10.1371/journal.pone.0355202)
Supplement: Supplementary file 1 [file pone.0355202.s001.docx]

**S1 Table. Baseline characteristics of the study population according to statin use (non-users, continuous users, and non-continuous users).**

| **Variable** | **Non-users  (n=1,966)** | **Continuous statin users  (n=334)** | **Non-continuous statin users  (n=388)** | **p-value** |
| --- | --- | --- | --- | --- |
| **Demographics** |  |  |  |  |
| Male sex, n (%) | 1104 (56.2) | 213 (63.8) | 263 (67.8) | **<0.001‡** |
| Female sex, n (%) | 862 (43.8) | 121 (36.2) | 125 (32.2) | **<0.001‡** |
| Age, years | 45.4 ± 9.9 | 54.7 ± 8.6 | 51.8 ± 9.1 | **<0.001†** |
| Height, cm | 168.5 ± 8.3 | 167.2 ± 8.6 | 168.8 ± 8.7 | **0.022†** |
| Weight, kg | 67.6 ± 13.2 | 70.0 ± 13.2 | 72.2 ± 13.6 | **<0.001†** |
| Body mass index, kg/m² | 23.6 ± 3.4 | 24.9 ± 3.3 | 25.2 ± 3.5 | **<0.001†** |
| **Body composition** |  |  |  |  |
| Body fat mass, kg | 18.7 ± 6.2 | 20.6 ± 6.2 | 21.0 ± 7.0 | **<0.001†** |
| Upper limb lean mass, kg | 5.12 ± 1.55 | 5.31 ± 1.49 | 5.56 ± 1.50 | **<0.001†** |
| Lower limb lean mass, kg | 15.56 ± 3.39 | 15.55 ± 3.49 | 16.15 ± 3.47 | **0.007†** |
| Appendicular lean mass, kg | 20.69 ± 4.86 | 20.87 ± 4.92 | 21.71 ± 4.91 | **0.001†** |
| Skeletal muscle index, kg/m² | 7.19 ± 1.18 | 7.36 ± 1.12 | 7.51 ± 1.10 | **<0.001†** |
| **Laboratory parameters** |  |  |  |  |
| Albumin, g/dL | 4.50 ± 0.25 | 4.61 ± 0.27 | 4.53 ± 0.26 | **<0.001†** |
| Total cholesterol, mg/dL | 200.0 ± 33.3 | 170.8 ± 41.7 | 205.0 ± 44.3 | **<0.001†** |
| Triglycerides, mg/dL | 102 (72–149) | 115 (85–162) | 132 (96–182) | **<0.001§** |
| HDL cholesterol, mg/dL | 55.9 ± 15.5 | 51.9 ± 12.5 | 50.7 ± 13.4 | **<0.001†** |
| LDL cholesterol, mg/dL | 127.2 ± 32.3 | 101.0 ± 38.8 | 136.0 ± 42.8 | **<0.001†** |
| HbA1c, % | 5.54 ± 0.57 | 6.14 ± 0.87 | 5.98 ± 0.94 | **<0.001†** |
| White blood cell count, ×10³/µL | 5.24 ± 1.47 | 5.69 ± 2.04 | 5.52 ± 1.56 | **<0.001†** |
| Hemoglobin, g/dL | 14.52 ± 1.47 | 14.60 ± 1.38 | 14.87 ± 1.38 | **<0.001†** |
| CRP, mg/dL | 0.062 (0.033–0.127) | 0.055 (0.029–0.110) | 0.078 (0.044–0.158) | **<0.001§** |
| **Questionnaire variables** |  |  |  |  |
| PHQ-9 score | 2 (0–5) | 1.5 (0–4) | 2 (0–5) | **0.012§** |
| GAD-7 score | 1 (0–4) | 0 (0–3) | 1 (0–3) | **0.007§** |
| Healthy dietary score | −7 (−10 to −4) | −5 (−8 to −2) | −7 (−10 to −3) | **<0.001§** |
| Current smoker, n (%) | 426 (21.7) | 77 (23.1) | 105 (27.1) | 0.066‡ |
| Alcohol consumption, n (%) | 1612 (82.0) | 261 (78.1) | 278 (71.6) | **<0.001‡** |
| Physical activity, n (%) |  |  |  | 0.630‡ |
| Level 1 | 1608 (81.8) | 275 (82.3) | 306 (78.9) |  |
| Level 2 | 310 (15.8) | 53 (15.9) | 72 (18.6) |  |
| Level 3 | 41 (2.1) | 5 (1.5) | 10 (2.6) |  |
| Level 4 | 7 (0.4) | 1 (0.3) | 0 (0) |  |
| **Comorbidities** |  |  |  |  |
| Heart disease, n (%) | 22 (1.1) | 13 (3.9) | 14 (3.6) | **<0.001‡** |
| Hypertension, n (%) | 189 (9.6) | 157 (47.0) | 120 (30.9) | **<0.001‡** |
| Diabetes mellitus, n (%) | 47 (2.4) | 97 (29.0) | 67 (17.3) | **<0.001‡** |
| Dyslipidemia, n (%) | 5 (0.3) | 182 (54.5) | 52 (13.4) | **<0.001‡** |

Physical activity was categorized into four ordinal levels, with Level 1 indicating the lowest and Level 4 the highest activity level.

Values are presented as the mean ± standard deviation or median (interquartile range), as appropriate.

† Continuous variables were compared using one-way analysis of variance (ANOVA) or Welch’s ANOVA when the assumption of equal variances was violated.

§ Skewed continuous variables were compared using the Kruskal–Wallis test.

‡ Categorical variables were compared using the chi-square or Fisher’s exact tests, as appropriate.
