# Supplementary material for: Association between statin use and longitudinal changes in skeletal muscle mass: A population-based cohort study
Source: PLoS One. 2026 Sep 3;21(9):e0355202. doi: 10.1371/journal.pone.0355202 (PMC13541165; doi:10.1371/journal.pone.0355202)
Supplement: Supplementary file 2 [file pone.0355202.s002.docx]

**S2 Table. Pairwise comparisons of baseline characteristics of the study population according to statin use (non-users, continuous users, and non-continuous users).**

| **Variable** | **Non-users vs Continuous statin users** | **Non-users vs Non-continuous statin users** | **Continuous statin users vs Non-continuous statin users** |
| --- | --- | --- | --- |
| **Demographics** |  |  |  |
| Sex | **0.009** | **<0.001** | 0.257 |
| Age, years | **<0.001** | **<0.001** | **<0.001** |
| Height, cm | **0.034** | 0.768 | **0.036** |
| Weight, kg | **0.005** | **<0.001** | 0.073 |
| Body mass index, kg/m² | **<0.001** | **<0.001** | 0.502 |
| **Body composition** |  |  |  |
| Body fat mass, kg | **<0.001** | **<0.001** | 0.538 |
| Upper limb lean mass, kg | 0.076 | **<0.001** | 0.065 |
| Lower limb lean mass, kg | 0.999 | **0.006** | 0.056 |
| Appendicular lean mass, kg | 0.819 | **0.001** | 0.058 |
| Skeletal muscle index, kg/m² | **0.045** | **<0.001** | 0.171 |
| **Laboratory parameters** |  |  |  |
| Albumin, g/dL | **<0.001** | 0.129 | **<0.001** |
| Total cholesterol, mg/dL | **<0.001** | 0.090 | **<0.001** |
| Triglycerides, mg/dL | **<0.001** | **<0.001** | **0.002** |
| HDL cholesterol, mg/dL | **<0.001** | **<0.001** | 0.438 |
| LDL cholesterol, mg/dL | **<0.001** | **<0.001** | **<0.001** |
| HbA1c, % | **<0.001** | **<0.001** | 0.056 |
| White blood cell count, ×10³/µL | **<0.001** | **0.003** | 0.443 |
| Hemoglobin, g/dL | 0.562 | **<0.001** | **0.022** |
| CRP, mg/dL | **0.040** | **<0.001** | **<0.001** |
| **Questionnaire variables** |  |  |  |
| PHQ-9 score | **0.010** | 0.062 | 0.524 |
| GAD-7 score | **0.005** | 0.066 | 0.327 |
| Healthy dietary score | **<0.001** | **0.035** | **0.001** |
| Current smoker | 0.571 | **0.020** | 0.216 |
| Alcohol consumption | 0.094 | **<0.001** | **0.045** |
| Physical activity | 0.912 | 0.303 | 0.364 |
| **Comorbidities** |  |  |  |
| Heart disease | **<0.001** | **<0.001** | 0.841 |
| Hypertension | **<0.001** | **<0.001** | **<0.001** |
| Diabetes mellitus | **<0.001** | **<0.001** | **<0.001** |
| Dyslipidemia | **<0.001** | **<0.001** | **<0.001** |

Data are presented as p-values for pairwise comparisons.

Continuous variables with normal distribution were analyzed using one-way ANOVA followed by Games–Howell post hoc tests.

Continuous variables with non-normal distribution were analyzed using the Mann–Whitney U test for pairwise comparisons.

Categorical variables were compared using the chi-square test or Fisher’s exact test, as appropriate.

P-values < 0.05 were considered statistically significant.

Chi-square tests were used, and results were interpreted with caution when expected cell counts were small.

P-values are presented without formal adjustment for multiple comparisons and should be interpreted with caution.
