# Supplementary material for: Association between statin use and longitudinal changes in skeletal muscle mass: A population-based cohort study
Source: PLoS One. 2026 Sep 3;21(9):e0355202. doi: 10.1371/journal.pone.0355202 (PMC13541165; doi:10.1371/journal.pone.0355202)
Supplement: Supplementary file 3 [file pone.0355202.s003.docx]

**S3 Table.** **Time-related changes in skeletal muscle outcomes and between-group differences based on statin use (linear mixed models, participants with follow-up ≥1 year; total n = 1789, statin users = 275, non-statin users = 1514).**

| **Outcome** | **Covariates** | **Statin users slope  (95% CI)** | **p for time** | **Δ slope (Non-statin − Statin) (95% CI)** | **p for interaction** | **Non-statin users slope** |
| --- | --- | --- | --- | --- | --- | --- |
| **Upper limb lean mass** | Unadjusted | −0.002 (−0.032 to 0.029) | 0.917 | +0.006 (−0.027 to 0.040) | 0.707 | +0.004 |
|  | Adjusted (age, sex) | +0.016 (−0.015 to 0.047) | 0.311 | +0.005 (−0.028 to 0.039) | 0.749 | +0.021 |
|  | Adjusted (age, sex, BMI, DM) | +0.025 (−0.004 to 0.055) | 0.093 | −0.006 (−0.038 to 0.026) | 0.703 | +0.019 |
| **Lower limb lean mass** | Unadjusted | −0.097 (−0.138 to −0.055) | <0.001 | +0.042 (−0.004 to 0.087) | 0.074 | −0.055 |
|  | Adjusted (age, sex) | −0.036 (−0.078 to 0.007) | 0.101 | +0.042 (−0.004 to 0.087) | 0.074 | +0.006 |
|  | Adjusted (age, sex, BMI, DM) | −0.021 (−0.063 to 0.020) | 0.308 | +0.024 (−0.020 to 0.068) | 0.288 | +0.003 |
| **Appendicular lean mass** | Unadjusted | −0.074 (−0.126 to −0.022) | 0.005 | +0.028 (−0.029 to 0.084) | 0.338 | −0.046 |
|  | Adjusted (age, sex) | +0.001 (−0.052 to 0.054) | 0.970 | +0.027 (−0.029 to 0.084) | 0.342 | +0.028 |
|  | Adjusted (age, sex, BMI, DM) | +0.026 (−0.022 to 0.075) | 0.289 | −0.003 (−0.055 to 0.049) | 0.903 | +0.023 |
| **Skeletal muscle index (SMI)** | Unadjusted | −0.016 (−0.043 to 0.010) | 0.220 | +0.007 (−0.022 to 0.036) | 0.641 | −0.009 |
|  | Adjusted (age, sex) | −0.007 (−0.034 to 0.019) | 0.599 | +0.006 (−0.023 to 0.035) | 0.679 | −0.001 |
|  | Adjusted (age, sex, BMI, DM) | +0.002 (−0.023 to 0.027) | 0.855 | −0.005 (−0.033 to 0.022) | 0.697 | −0.003 |

Values are presented as β coefficients with 95% confidence intervals derived from linear mixed models with random intercepts for participants.

Three models are presented: an unadjusted model; a model adjusted for age and sex; and a fully adjusted model including age, sex, body mass index, and diabetes mellitus.

β coefficients represent estimated annual rate of change (slope) in each outcome.

Statin users served as the reference group.

The time coefficient represents rate of change over time in statin users.

Δ slope represents the between-group difference in the rate of change (non-statin minus statin).

Non-statin users slope is presented as a point estimate derived from the linear combination of fixed-effect coefficients.

A positive Δ slope indicates a less steep decline (or relative preservation) among non-statin users compared with statin users.

All models are presented within a single table for clarity and compact comparison.
