# Supplementary material for: Association between statin use and longitudinal changes in skeletal muscle mass: A population-based cohort study
Source: PLoS One. 2026 Sep 3;21(9):e0355202. doi: 10.1371/journal.pone.0355202 (PMC13541165; doi:10.1371/journal.pone.0355202)
Supplement: Supplementary file 4 [file pone.0355202.s004.docx]

**S4 Table.** **Time-related changes in skeletal muscle outcomes and between-group differences based on statin use (linear mixed models, participants aged ≥60 years; total n = 189, statin users = 77, non-statin users = 112).**

| **Outcome** | **Covariates** | **Statin users slope  (95% CI)** | **p for time** | **Δ slope (Non-statin − Statin) (95% CI)** | **p for interaction** | **Non-statin users slope** |
| --- | --- | --- | --- | --- | --- | --- |
| **Upper limb lean mass** | Unadjusted | −0.028 (−0.090 to 0.034) | 0.377 | +0.048 (−0.030 to 0.127) | 0.227 | +0.020 |
|  | Adjusted (age, sex) | −0.000 (−0.064 to 0.064) | 0.994 | +0.048 (−0.030 to 0.126) | 0.227 | +0.048 |
|  | Adjusted (age, sex, BMI, DM) | +0.007 (−0.055 to 0.070) | 0.814 | +0.044 (−0.033 to 0.122) | 0.257 | +0.051 |
| **Lower limb lean mass** | Unadjusted | −0.116 (−0.237 to −0.095) | <0.001 | +0.068 (−0.022 to 0.158) | 0.137 | −0.048 |
|  | Adjusted (age, sex) | −0.095 (−0.177 to −0.013) | 0.023 | +0.069 (−0.021 to 0.158) | 0.133 | −0.026 |
|  | Adjusted (age, sex, BMI, DM) | −0.076 (−0.154 to 0.003) | 0.060 | +0.056 (−0.031 to 0.143) | 0.203 | −0.020 |
| **Appendicular lean mass** | Unadjusted | −0.130 (−0.216 to −0.043) | 0.004 | +0.085 (−0.025 to 0.195) | 0.131 | −0.045 |
|  | Adjusted (age, sex) | −0.033 (−0.136 to 0.071) | 0.535 | +0.086 (−0.025 to 0.196) | 0.127 | +0.053 |
|  | Adjusted (age, sex, BMI, DM) | −0.003 (−0.103 to 0.096) | 0.947 | +0.067 (−0.042 to 0.176) | 0.228 | +0.064 |
| **Skeletal muscle index (SMI)** | Unadjusted | −0.067 (−0.121 to −0.013) | 0.016 | +0.067 (−0.001 to 0.135) | 0.054 | +0.000 |
|  | Adjusted (age, sex) | −0.041 (−0.094 to −0.011) | 0.124 | +0.057 (−0.007 to 0.121) | 0.082 | +0.016 |
|  | Adjusted (age, sex, BMI, DM) | −0.027 (−0.078 to 0.025) | 0.306 | +0.045 (−0.018 to 0.109) | 0.161 | +0.018 |

Values are presented as β coefficients with 95% confidence intervals derived from linear mixed models with random intercepts for participants.

Three models are presented: an unadjusted model; a model adjusted for age and sex; and a fully adjusted model including age, sex, body mass index, and diabetes mellitus.

β coefficients represent estimated annual rate of ch ange (slope) in each outcome.

Statin users served as the reference group.

The time coefficient represents rate of change over time in statin users.

Δ slope represents the between-group difference in the rate of change (non-statin minus statin).

Non-statin users slope is presented as a point estimate derived from the linear combination of fixed-effect coefficients.

A positive Δ slope indicates a less steep decline (or relative preservation) among non-statin users compared with statin users.

All models are presented within a single table for clarity and compact comparison.
